# *Peste des petits ruminants* virus infection of Black Bengal goats showed altered haematological and serum biochemical profiles

**DOI:** 10.4102/ojvr.v85i1.1595

**Published:** 2018-10-15

**Authors:** Shahana Begum, Mohammed Nooruzzaman, Murshida Parvin, Nijaya Mohanto, Rokshana Parvin, Mohammad R. Islam, Emdadul H. Chowdhury

**Affiliations:** 1 Department of Pathology, Bangladesh Agricultural University, Bangladesh

## Abstract

In Bangladesh, veterinarians often claim to reduce the mortality of natural *peste des petits ruminants* (PPR) outbreaks with the help of supportive fluid and electrolyte therapy. Information on haematological and biochemical parameters of PPR-infected goats, which is often altered because of associated tissue damages, is necessary to formulate the appropriate supportive therapy. This study determined the haematological and serum biochemical parameters of Black Bengal goats naturally infected with PPR virus. Blood and serum samples from 13 PPR-affected Black Bengal goats from 13 field outbreaks and 5 healthy goats were collected and analysed by routine haematological and biochemical examination. Haematological analysis of PRR-affected goats showed severe anaemia characterised by significant decrease in the values of haemoglobin, total erythrocyte counts (TECs) and packed cell volume (PCV). On the contrary, PPR-affected goats showed marked leucocytosis with absolute increase in lymphocytes and neutrophils counts compared to the healthy goats. Biochemical analysis revealed significant decrease in total protein and albumin level and increased creatine kinase, aspartate transaminase and alanine transaminase that mirrored the gross and histopathological changes in the PPR-affected goats. Significant increase in the values of sodium and chloride ions was found in the sera of PPR-infected goats. *Peste des petits ruminants* virus altered the haematological and serum biochemical parameters of the infected goats. Antidiarrheal agents with aqua solution together with other drugs to support liver and kidney function could help improve therapy of PPR-infected goats.

## Introduction

*Peste des petits ruminants* (PPR) is an acute, highly contagious viral disease of goats, sheep and other related species. *Peste des petits ruminants* is one of the main transboundary animal diseases and presents a major threat to animal production in PPR endemic areas because of high case fatality reaching 90% in native animals and also because of trade restriction (Diallo 2003). The disease is caused by the PPR virus (PPRV) which is a single-stranded negative sense ribonucleic acid (RNA) virus under the genus *Morbillivirus*, family Paramyxoviridae and antigenically related to the rinderpest, canine distemper and measles virus (Gibbs et al. 1979). The virus is circulating in Africa, the Middle East and Central to South-East Asia in four genetically distinct lineages, three of which (I, II, III) were first described in Africa, including Guinea, Ivory Coast, Senegal, Mali, Burkina Faso, Ghana, Nigeria, Uganda and Tanzania, and the fourth one (IV) in Asia (Banyard et al. 2010; Chowdhury et al. 2014; Rahman et al. 2016). However, the Asian lineage was recently introduced in some countries of Africa and Europe, indicating continuing spread of the virus across the continents (Maganga et al. 2013; Parida et al. 2016).

The first outbreak of PPR in Bangladesh was recorded in 1993 (Islam et al. 2001). Since then the outbreaks of PPR are being recorded regularly across the country (Chowdhury et al. 2014; Bhuiyan et al. 2014; Rahman et al. 2011, 2016). Clinical PPR outbreaks are often marked by high fever, mucopurulent discharges from nose, eyes and mouth; severe diarrhoea; enteritis; bronchopneumonia and erosive stomatitis with high morbidity and mortality rates (Bhuiyan et al. 2014). In the majority of PPR outbreaks, average flock morbidity and mortality of 75% and 59%, respectively, and a case fatality rate of 74% were recorded (Chowdhury et al. 2014). In 2010, there were approximately 84 000 hospital cases of PPR in Bangladesh, with an estimated direct annual loss of Tk1842 million (US$24.56) (Siddiky 2013). Lack of quality vaccines together with poor presumptive diagnosis hindered the success of PPR control programmes (Haider et al. 2017). Veterinarians often claim to reduce the mortality of clinical PPR outbreaks to some extent with the help of supportive therapy. Moreover, a recent study shows that supportive therapy in the form of fluid and antibiotics combined with PPR-specific hyperimmune serum can reduce the mortality in PPR-infected goats (Yousuf et al. 2015). However, the knowledge of haematological, serum biochemical and electrolyte profiles of clinically PPR-infected goats, which is often altered because of diarrhoea and mucopurulent discharge, is needed to better describe the disease status and formulate appropriate supportive therapy. Therefore, this study was designed to assess the serum biochemical and haematological profiles of PPR-infected Black Bengal goats in Bangladesh.

## Materials and methods

### Animals

A total of 13 flocks of Black Bengal goats with suspected PPR outbreaks were selected from two villages in the Mymensingh district of Bangladesh during the period July 2015 to June 2016. The detailed descriptions of the flocks are shown in Table 1. Blood and nasal swab were collected from one PPR-infected goat of each flock that showed clinical signs of PPR. In addition, blood samples were collected from five apparently healthy goats that served as controls. The five healthy goats were obtained from PPR seronegative flocks other than the 13 infected flocks. All animal handling, necropsy and sample collection was performed by trained veterinarians. The study was carried out in accordance with the recommendation of the Animal Experimentation Ethics Committee of Bangladesh Agricultural University, Mymensingh (ref. no.: AEEC/01/2015). The protocol was approved by the Animal Experimentation Ethics Committee. Prior approval was obtained from the animal owners regarding further use of the initially collected diagnostic samples.

**TABLE 1:** Morbidity and mortality of Black Bengal goats naturally infected with *peste des petits ruminants* virus.

Flock no.	Flock size	Age range of flock (months)	Age of sampled animals (months)	Sex of sampled animals	No. of affected animals	No. of dead animals	Body temperature	Flock morbidity (%)	Flock mortality (%)	Case fatality rate (%)	RT-PCR positive blood (B) or nasal swabs (NS)	No. of necropsy
1	14	2–24	18	Male	14	10	105.0 ^o^F	100.00	71.43	71.43	NS	01
2	16	6–36	12	Female	12	12	105.0 ^o^F	75.00	100.00	75.00	NS	01
3	9	3–24	18	Male	7	5	105.0 ^o^F	77.77	71.43	55.56	NS	Not performed
4	9	4–18	4	Female	8	7	105.0 ^o^F	88.88	87.50	77.77	B & NS	01
5	12	6–24	18	Female	10	5	106.0 ^o^F	83.33	50.00	41.66	S	01
6	8	3–18	3	Female	5	2	106.0 ^o^F	62.50	40.00	25.00	NS	01
7	8	2–36	4	Female	4	2	106.0 ^o^F	50.00	50.00	25.00	B & NS	01
8	5	6–18	6	Female	5	3	105.0 ^o^F	100.00	60.00	60.00	NS	01
9	10	3–30	4	Male	6	4	106.0 ^o^F	60.00	66.67	40.00	B & NS	Not performed
10	6	2–24	6	Male	6	2	105.0 ^o^F	100.00	33.33	33.33	NS	Not performed
11	8	8–24	12	Male	5	3	104.8 ^o^F	62.5	60.00	37.50	B & NS	Not performed
12	7	4–30	18	Female	6	5	105.0 ^o^F	85.71	83.33	71.43	B & NS	Not performed
13	5	4–36	12	Male	5	4	105.0 ^o^F	100.00	80.00	80.00	NS	Not performed
Total	125	-	-	-	99	70	-	80.44	65.67	53.36	-	-

No., number; RT-PCR, reverse transcription-polymerase chain reaction; B, blood; NS, nasal swab.

### Necropsy and histopathology

A total of seven dead goats were collected from seven PPR-suspected flocks and routine necropsy was performed in the Department of Pathology. Gross pathological changes were recorded. The tissue samples of lips, trachea, lungs, spleen, lymph node, intestine, kidney, heart, spleen and liver were collected in 10% neutral buffered formalin and processed for histopathological study following standard procedures (Luna 1988). In addition, bronchial and mesenteric lymph nodes were collected aseptically and stored at -80 ^o^C for molecular detection of the virus.

### Molecular detection of the *peste des petits ruminants* virus

Blood and nasal swabs from PPR-suspected sick goats were soaked onto small pieces of filter paper as described elsewhere (Bhuiyan et al. 2014; Forsyth & Barrett 1995). In brief, a few drops of blood were poured on the filter papers, from the base towards the tip, until it was completely soaked. Nasal swab were collected with sterilised filter paper which was directly applied on the nostrils of PPR-suspected goats. The filter papers were air-dried avoiding direct sunlight and stored at -70 ^o^C in screw-capped tubes. The smeared filter papers were directly used in polymerase chain reaction (PCR) tubes as a source of RNA template as described previously (Bhuiyan et al. 2014). In addition, RNA was extracted from 20% tissue homogenate of bronchial and mesenteric lymph nodes of PPR-suspected dead goats using RNeasy Mini Kit (Qiagen, Germany). The presence of PPR virus was detected by an established reverse transcription-PCR (RT-PCR) technique (Forsyth & Barret 1995) to amplify a 448 bp fragment of the F gene. The RT-PCR was performed with Qiagen One-Step RT-PCR kit (Qiagen, Germany) as per the manufacturer’s instruction.

### Competitive enzyme-linked immunosorbent assay

Approximately 7 mL of blood per goat was collected aseptically from the jugular vein of 13 PPRV RT-PCR positive goats after 10–14 days of onset of clinical signs. In addition, blood samples from five healthy goats were collected as control. After collection, 2 mL of blood was transferred to a sterile vial containing ethylenediaminetetraacetic acid (EDTA) at the rate of 1 mg/mL blood for routine examination of blood. Approximately 5 mL of blood was transferred to another sterile tube without EDTA for serum separation. After clotting of the blood, serum samples were obtained by centrifugation at 900× *g* for 10 min and stored at -20 ^°^C for serological and biochemical analysis. The amount of PPRV nucleoprotein-specific antibody in serum samples was quantified by a competitive enzyme-linked immunosorbent assay (ELISA) kit (ID Screen PPR Competition, ID-Vet, Montpellier, France).

### Haematological analysis

Routine haematological examination of whole blood samples was performed by standard method to determine the values of haemoglobin (Hb), erythrocyte sedimentation rate (ESR), packed cell volume (PCV), total erythrocyte count (TEC), total leucocyte count (TLC) and differential leucocyte count (DLC) (Lamberg & Rothstein 1978).

### Biochemical analysis

For determination of serum electrolytes (sodium, potassium and chloride ions), samples were analysed from a commercial lab (Pure Diagnostic Center, Mymensingh, Bangladesh) with an automated electrolyte analyzer GENLYTE 3000A (IVD) as per kit instruction (Electrolyte solution, Biogen, GmbH, Germany). Other serum biochemical constituents such as inorganic phosphorus (P), calcium (Ca), total protein (TP), albumin, glucose, bilirubin and blood urea nitrogen (BUN), creatine kinase (CK), alkaline phosphatase (ALP), alanine transaminase (ALT) and aspartate transaminase (AST) were determined with the help of an automated T80 Ultraviolet–visible spectroscopy (UV/VIS) spectrophotometer (PG Instruments, UK) at Bangladesh Agricultural University Central Laboratory, Mymensingh, Bangladesh.

### Statistical analysis

Data were statistically analysed using the software package GraphPad Prism Version 5.0. Differences in haematological and biochemical profiles between PPRV-infected and healthy goats were evaluated statistically by one-tailed non-parametric Mann–Whitney U test. A *p* value of ≤ 0.05 was considered significant.

## Results

### Outbreak investigation

A total of 13 PPR-suspected flocks of Black Bengal goats were investigated in the present study. Detailed description of the affected flocks is given in Table 1. Out of 125 goats examined, 99 showed clinical signs of PPR such as high fever (105 °F), profuse nasal and ocular discharge and severe diarrhoea; 70 goats died within 7–12 days after onset of clinical signs. Therefore, the average morbidity and mortality of the PPR-suspected flocks were 80.44% and 65.67%, respectively. The case fatality varied from 25% to 100%, with an average of 56.36%. It is noteworthy that none of the examined animals received any supportive therapy.

### Molecular detection of the virus

The blood and nasal swabs from 13 PPR-suspected goats were collected on small pieces of filter paper and analysed by RT-PCR to amplify a 488 bp fragment of F gene of PPR virus. The filter paper RT-PCR method successfully detected PPRV in all of the tested nasal swabs. However, blood samples from only five PPR-suspected goats produced a positive result in the RT-PCR. It is noteworthy that goats that were positive with nasal swabs, but negative with blood were sampled at much later stages of fever. In addition, the RT-PCR method successfully amplified PPRV in lymph node samples collected during necropsy from PPR-suspected goats.

### Pathological investigation

**FIGURE 1:**
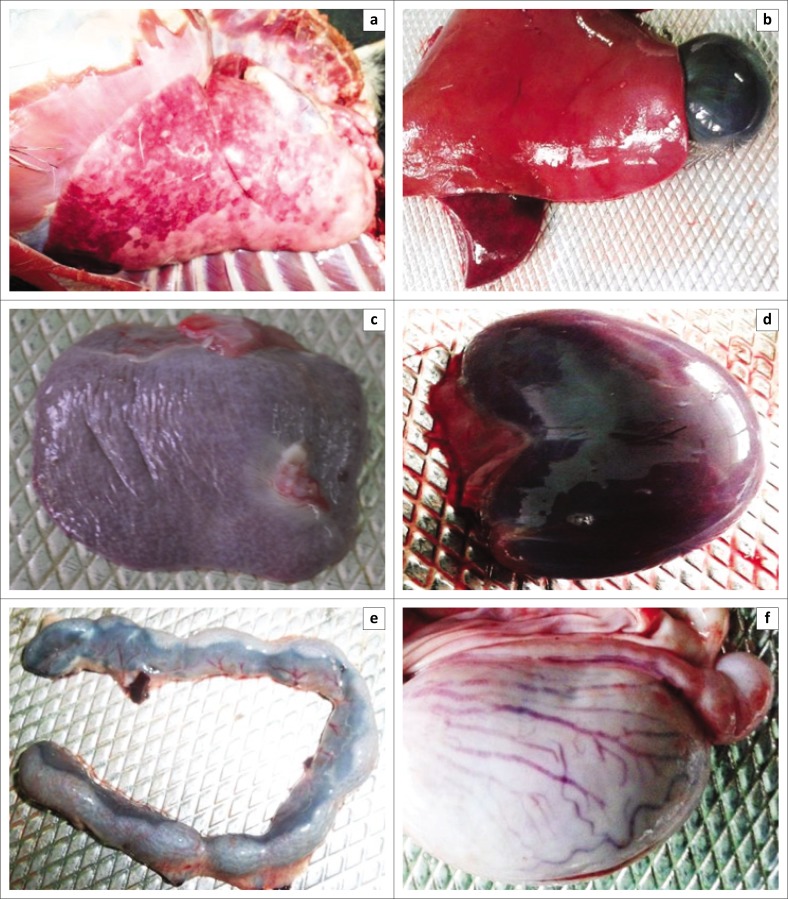
Post-mortem examinations of *peste des petits ruminants*–infected Black Bengal goats showed severe haemorrhage and congestion of visceral organs. (a) Lungs showed severe congestion with white necrotic foci on the surface. (b) Congested and fragile liver and (c) atrophy of the spleen. (d) Kidneys showed congestion and (e) mesenteric lymph nodes were enlarged and congested. (f) Congestion and swelling of testis were found in *peste des petits ruminants*–infected male goat. Representative images of one out of seven dead goats are shown.

Erosive and necrotic spots on the lip and tongue with severely congested trachea were observed in the dehydrated carcasses. Significant lesions were found in the lungs, which included congestion and consolidation, mostly in the apical lobes, together with white necrotic foci on the surface (Figure 1a). Congestion and fragility of the liver (Figure 1b) and atrophy of the spleen (Figure 1c) were found. The kidney (Figure 1d) and the mesenteric lymph nodes (Figure 1e) were enlarged two to three times and showed congestion. Haemorrhagic streaks were seen in the duodenum and the terminal ileum and severe congestion was also observed in the testicles of two male goats (Figure 1f).

**FIGURE 2:**
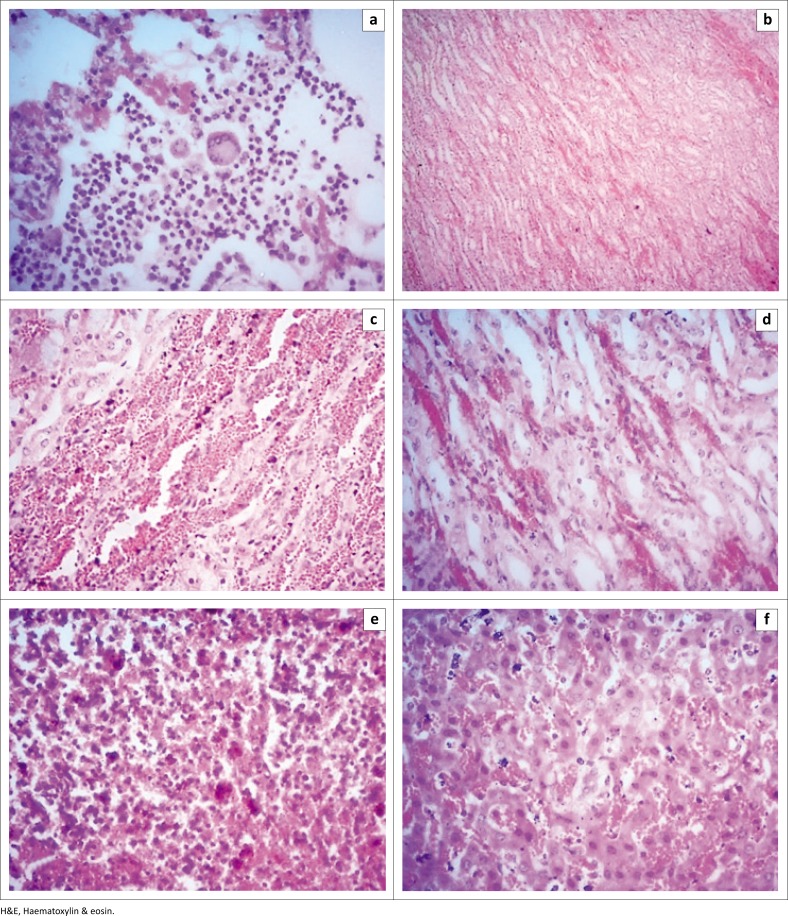
Histopathological findings of *peste des petits ruminants*–infected Black Bengal goats showed haemorrhage and mononuclear infiltration. (a) *Peste des petits ruminants*–infected lungs showed clumps of large mononuclear and multinucleated cells within the alveoli (H&E stain, 250×). (b) Kidney showed haemorrhage in the tubular area (H&E stain; 62.5×). (c) and (d) Kidneys showed severe haemorrhages and congestion with haemosiderosis (H&E stain, 250×). (e) Haemosiderosis with severe congestion in the spleen (H&E stain, 250×). (f) Congestion with stagnant of bile pigments in the liver (H&E stain, 250×).

The histopathological changes in the lungs, kidney and lymph nodes of PPR-infected goats were marked by severe haemorrhage and congestion. The affected lungs showed syncytial cell formation in the alveoli and clumps of mononuclear cells infiltration in the inter-alveolar space and bronchial wall (Figure 2a). Massive haemorrhage in the tubular areas of kidneys was found in some goats (Figure 2b). Severe haemorrhage and congestion with brown colour haemosiderin deposition were found in kidneys (Figures 2c & 2d). The spleen was congested with haemosiderin deposition (Figure 2e). Severe congestion and haemorrhage with stagnation of bile pigments were found in the liver parenchyma (Figure 2f). Taken together, natural PPR infections of Black Bengal goats showed severe haemorrhage and congestion in different visceral organs.

### Haematological profiles of naturally *peste des petits ruminants–*infected goats

**FIGURE 3:**
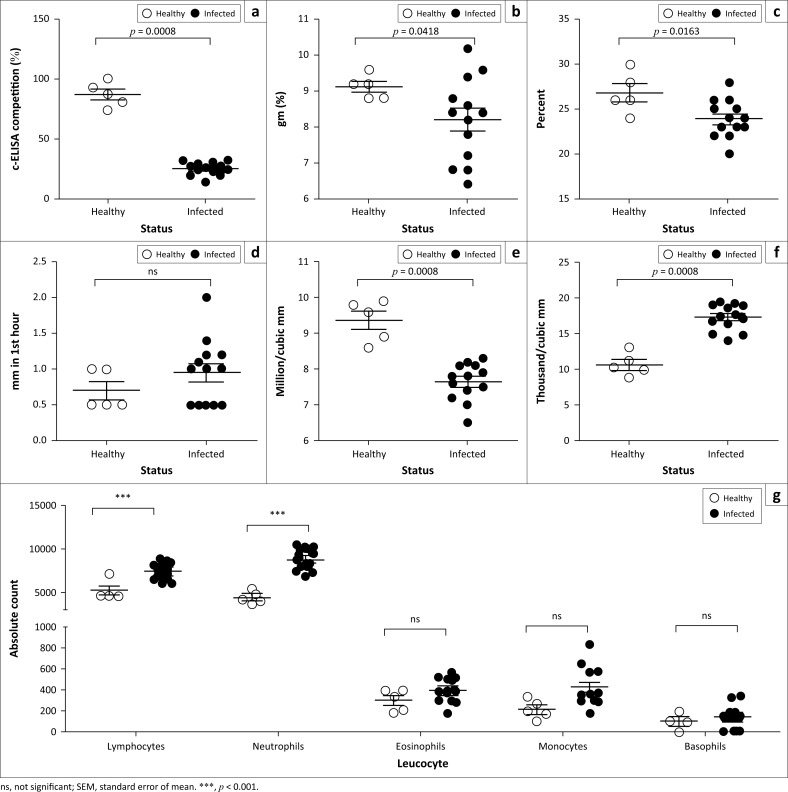
Haematological profile of *peste des petits ruminants* virus–infected Black Bengal goats showed marked anaemia and leucocytosis. Blood samples were collected from healthy and *peste des petits ruminants*–affected goats and the amount of (a) *peste des petits ruminants* virus–specific antibody, (b) haemoglobin, (c) packed cell volume, (d) erythrocyte sedimentation rate, (e) total erythrocyte counts, (f) leucocyte count and (g) differential leucocyte count were quantified. Data indicate mean ± SEM from 5 healthy and 13 *peste des petits ruminants* virus–infected goats. One-tailed Mann–Whitney test. *p* ≤ 0.05 was considered statistically significant.

To investigate the haematological profiles of goats with natural PPR outbreaks, serum samples were collected from 13 infected and 5 healthy Black Bengal goats. The serum samples were first screened for the presence of PPRV-specific antibodies by a competitive ELISA. All serum samples from PPR-suspected goats showed a high level of PPRV-specific antibody when goats survived 10 days after onset of clinical signs (Figure 3a). The control goats did not show any PPRV-specific antibody response. Then, we performed routine examination of whole blood samples from PPR-infected as well as healthy goats. There was a significant decrease in the values of Hb concentration (Figure 3b), PCV (Figure 3c) and TEC (Figure 3e) in the PPR-infected goats. On the other hand, TLC (Figure 3f) was significantly increased in PPR-infected goats as compared to healthy animals. Further analysis of the DLC showed a significant increase in the absolute number of lymphocytes and neutrophils (Figure 3g). However, the other blood parameters such as ESR (Figure 3d) and absolute monocytes, eosinophils and basophils counts (Figure 3g) remained comparable between PPR-infected and healthy goats. Collectively, natural PPR infection in goats induced severe anaemia and leucocytosis.

### Serum biochemical analysis of natural *peste des petits ruminants* virus–infected goats

**FIGURE 4:**
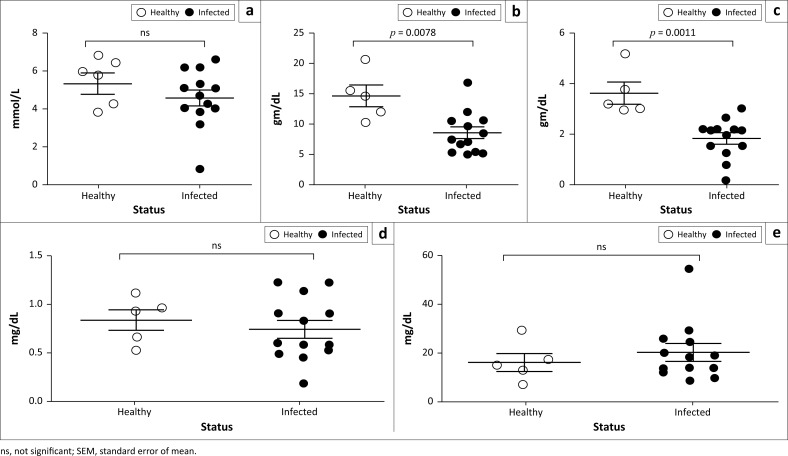
*Peste des petits ruminants* virus–infected Black Bengal goats showed altered serum biochemical profiles. Serum samples were collected from healthy and *peste des petits ruminants*–affected goats and the level of (a) glucose, (b) total protein, (c) albumin, (d) bilirubin and (e) blood urea nitrogen was quantified. Data indicate mean ± SEM from 5 healthy and 13 *peste des petits ruminants* virus–infected goats. One-tailed Mann–Whitney test. *p* ≤ 0.05 was considered statistically significant.

**FIGURE 5:**
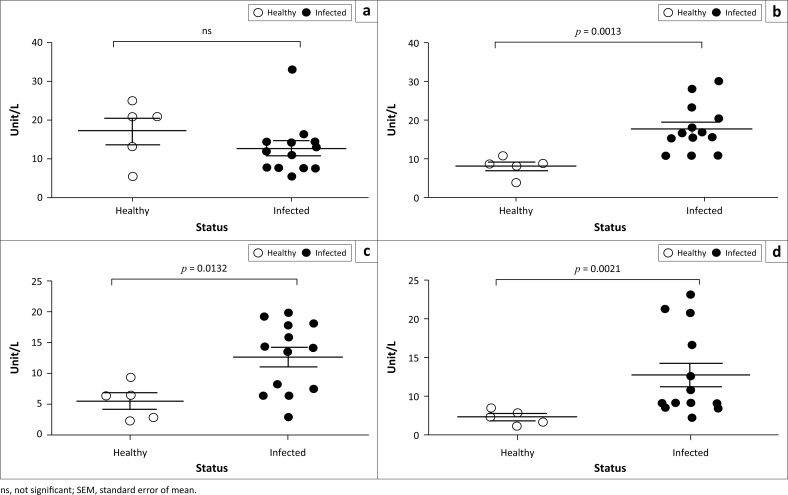
Natural *peste des petits ruminants* virus–infected Black Bengal goats showed elevated serum enzymes. Serum samples were collected from healthy and *peste des petits ruminants*–affected goats and the amount of (a) alkaline phosphatase, (b) creatine kinase, (c) aspartate transaminase and (d) alanine transaminase was measured. Data indicate mean ± SEM from 5 healthy and 13 *peste des petits ruminants* virus–infected goats. One-tailed Mann–Whitney test. *p* ≤ 0.05 was considered statistically significant.

Different biochemical parameters of serum samples obtained from PPR-infected goats were quantified and compared with healthy goats. *Peste des petits ruminants*–infected goats showed significantly lower level of total protein (Figure 4b) and albumin (Figure 4c) in their sera compared to healthy goats. No significant difference was found in the amount of glucose (Figure 4a), bilirubin (Figure 4d) and BUN (Figure 4e) between PPR-infected and healthy goats. Further analysis of the serum enzymes showed a significant increase in the level of CK (Figure 5b), AST (Figure 5c) and ALT (Figure 5d) in the PPR-infected goats compared to the healthy goats. However, the amount of ALP remained constant between the infected and healthy goats.

**FIGURE 6:**
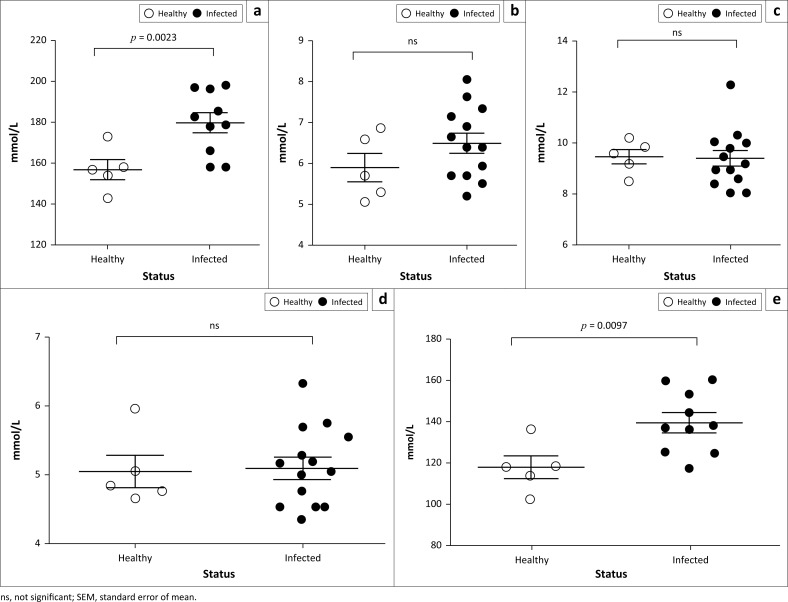
Serum electrolytes analysis of natural *peste des petits ruminants* virus–infected Black Bengal goats showed elevated level of sodium and chloride ions. Serum samples were collected from healthy and PPR-affected goats and the values of (a) sodium, (b) potassium, (c) calcium, (d) phosphorus and (e) chloride were quantified. Data indicate mean ± SEM from 5 healthy and 10–13 *peste des petits ruminants* virus–infected goats. One-tailed Mann–Whitney test. *p* ≤ 0.05 was considered statistically significant.

Furthermore, we determined the concentration of some important serum electrolytes in PPR-infected and healthy goats. We found significantly higher levels of sodium (Figure 6a) and chloride (Figure 6e) ions in sera of PPR-infected goats than healthy goats. However, other electrolytes such as potassium, calcium and phosphorus level remained normal in both animal groups (Figures 6b, 6c & 6d). Of note, among infected animals, five goats with a very high level of sodium and chloride ions were suffering from severe diarrhoea and showed early mortality. Collectively, goats with natural PPRV infection showed an abnormal serum biochemical profile marked by hypoproteinaemia, increased kidney and liver enzymes and higher sodium and chloride ions.

## Discussion

*Peste des petits ruminants* virus infection induced marked anaemia, leucocytosis and altered serum enzyme and electrolyte profiles in Black Bengal goats. Outbreak investigation showed that all 13 goats included in the study were naturally infected with PPRV. Pathological investigation of all PPR-suspected goats showed marked bronchopneumonia and haemorrhagic lesions in different visceral organs that were typical of natural PPRV outbreaks (Chowdhury et al. 2014; Rahman et al. 2011, 2016). However, the haemorrhagic change in kidney was not reported earlier.

In Bangladesh, veterinarians generally treat PPR-infected goats with antibiotic, antihistaminic, dextrose saline and astringent mixture (Chowdhury et al. 2014). Veterinarians often claim to reduce mortality in infected flocks by controlling secondary bacterial infections and counteracting shock. However, information on the haemato-biochemical profiles of PPR-infected goats, which are often altered because of viremia and associated tissue destruction, is necessary to formulate an appropriate supporting therapy against devastating clinical PPR outbreaks. All serum samples from the confirmed PPR outbreaks showed PPRV-specific antibody titres during the later stage of the disease, particularly if the goat survived more than 10 days after onset of clinical signs. Thus, supportive therapy could provide a critical window for the development of adaptive immunity in infected goats during the first 10–12 days of infection. The routine haematological examination of the PPR-infected goats revealed a significant reduction in the values of Hb, PCV and TECs compared to healthy goats which was because of widespread haemorrhages throughout the body. A significant decrease in the values of Hb and erythrocyte count was also reported earlier (Das et al. 2015; Sharma et al. 2012) and expected in PPR-infected goats because of the massive haemorrhage in different visceral organs which we also observed during pathological investigations. We found an increase in the absolute number of lymphocytes and neutrophils in the PPR-infected goats. Similar findings of the leucocytosis in the PPR-infected goats were shown by some other studies indicating acute inflammation in the studied animals (Das et al. 2015; Ezeasor, Emikpe & Anosa 2015; Maina, Gitao & Gathumbi 2015). Although others reported some degree of leucopoenia in the PPR-infected goats (Sharma et al. 2012), such differences between research groups could possibly be explained by a variation in the disease status as well as nutritional condition of the infected animals. A decrease in total protein and albumin indicates liver and kidney dysfunction, which was mirrored by the gross and histopathological and biochemical findings of the infected goats. On the other hand, the level of important enzymes such as CK, AST and ALT increased significantly in the PPR-infected goats that correlated with the damage in liver and kidney by the virus (Al-Dubaib 2009; Chowdhury et al. 2014; Kul et al. 2007).

Diarrhoea together with an increased nasal, oral and ocular discharge was shown to produce severe dehydration and death in the PPR-infected animals (Chowdhury et al. 2014). In addition, pathological changes in the liver and kidney might lead to the fluid and electrolyte imbalance in the infected goats. We found a significantly higher concentration of sodium and chloride ions in the blood of PPR-infected goats than that of the healthy goats. Other electrolytes such as potassium, calcium and phosphorus remained unaltered. Increased sodium and chloride ions in PPR-infected goats were also found in similar other studies (Das et al. 2015; Kataria, Kataria & Gahlot 2007). Our study also found an association of the increased sodium and chloride level with the mortality in five severely diarrheic goats. In addition, the comparable level of glucose in the infected as well as healthy goats denied the necessity of applying dextrose saline as supportive therapy which is being used in the field to treat PPR-infected goats.

## Conclusion

In conclusion, haematological analysis of PPR-affected goats showed severe damage in the liver and kidneys, with marked anaemia, leucocytosis and altered serum biochemical profiles. The significant kidney lesions and increased level of CK highlight an increased tropism of the virus towards kidneys. Therefore, veterinarians should suggest antidiarrheic therapy in the form of oral or intravenous application of isotonic aqua solution together with other supportive drugs to support liver and kidney function for the treatment of natural PPR outbreaks. A clinical scoring system can be devised and applied in the field with a treatment plan informed by the haematological and serum biochemical profiles. However, it requires a further comprehensive study.
